# Effects and Progress of Photo-Crosslinking Hydrogels in Wound Healing Improvement

**DOI:** 10.3390/gels8100609

**Published:** 2022-09-23

**Authors:** Hao Ma, Yuan Peng, Shunuo Zhang, Yixin Zhang, Peiru Min

**Affiliations:** Department of Plastic and Reconstructive Surgery, Shanghai Ninth People’s Hospital Affiliated to Shanghai Jiao Tong University School of Medicine, Shanghai 200001, China

**Keywords:** hydrogels, photo-crosslinking, wound healing

## Abstract

Wound healing is a dynamic physiological process, including three stages: inflammation, tissue formation, and remodeling. The quality of wound healing is affected by many topical and systemic factors, while any small factor may affect the process. Therefore, improving the quality of wound healing is a complex and arduous challenge. Photo-crosslinking reaction using visible light irradiation is a novel method for hydrogel preparation. Photo-crosslinking hydrogels can be controlled in time and space, and are not interfered by temperature conditions, which have been widely used in the fields of medicine and engineering. This review aims to summarize the application of photo-crosslinking hydrogels in improving the quality of wound healing, mainly including the material design, application mechanism, and effect of photo-crosslinking hydrogels applied in wound healing, followed by the applicable animal models for experimental research. Finally, this review analyzes the clinical application prospects of photo-crosslinking hydrogels in the field of wound healing.

## 1. Introduction

The repair of wounds is one of the most complex biological processes that occur in human life [1]. Wound healing is a dynamic and complex physiological progress. After an injury occurs, various intracellular and intercellular pathways, cellular components of the immune system, the blood coagulation cascade, and the inflammatory pathways are activated if tissue integrity is to be restored [1,2]. Generally, wound healing contains three overlapping-in-time but different stages: inflammation, tissue formation, and tissue remodeling, involving the collaborative interactions among bioactive factors, the extracellular matrix (ECM), and a variety of cell lineages (including inflammatory cells, endothelial cells, fibroblasts, keratinocytes and blood cells) [1,3].

In the first stage of wound healing, inflammation occurs immediately after tissue damage and requires components of the coagulation cascade, inflammatory pathways, and immune system to stop bleeding and remove dead and devitalized tissues [4,5]. The main cells involved are platelets, neutrophils, and macrophages. Granulation tissue consisting of macrophages, fibroblasts, and endothelial cells is the hallmark of the proliferative phase, in addition to which re-epithelialization is essential to restore tissue integrity [5]. Following robust proliferation and ECM synthesis, wound healing enters the final remodeling phase, which can last for years [6]. In this phase, ECM can remodel to an architecture that approaches that of the normal tissue, and the wound edge also undergoes physical contraction mediated by myofibroblasts. More critically, the formation of granulation tissue ceases through apoptosis of the responsible cells, and its aberration leads to hypertrophic scarring and keloids [7].

Wound healing is affected by multiple factors, and impaired wound healing may be manifested as delayed or excessive healing. The factors affecting wound healing can be divided into topical and systemic factors. Topical factors are factors that directly affect wound characteristics, while systemic factors are overall health or disease states that affect individual healing ability [6]. Topical factors mainly include oxygenation [8,9], infection [10,11], foreign body [12]. Systemic factors include age and gender [13,14], sex hormones [15,16], stress [17], diseases (diabetes, fibrotic diseases, keloids, etc.) [18], obesity [19], medications (glucocorticoid steroids, nonsteroidal anti-inflammatory drugs, chemotherapy) [20,21,22], alcoholism and smoking [23,24], nutrition [25], and so on. Any small factor change may affect the process of wound healing. Therefore, improving the quality of wound healing is a complex and arduous challenge.

Hydrogels are an important class of biomaterials in the field of biology and medicine. Because of their hydrophilic characteristics, easy preparation, modification, and functionalization, hydrogels have been widely used as dressings in wound healing [26,27,28]. Hydrogels, composed of 3D network structures that mimic the extracellular matrix microenvironment, can maintain a moist microenvironment, absorb inflammatory exudates, and provide a physical barrier to prevent bacteria from entering the wound [29,30,31,32]. At the same time, a hydrogel matrix made of natural materials or synthetic polymers can be loaded with drugs or various bioactive substances, thus playing a variety of different roles in wound healing, such as antibacterial, anti-inflammatory, antioxidant, and tissue remodeling [27,33]. However, the effect of traditional hydrogels in wound healing is not perfect. In the field of surgery, we often encounter many wounds that are difficult to treat with hydrogel dressings. Traditional hydrogels exposed problems, such as inconvenient operation, short dwell time, and unstable biofixation when dealing with those wounds with an uneven or irregular shape and postoperative adhesion, resulting in a poor wound healing effect [34,35,36]. Therefore, it is still necessary to develop more ideal hydrogel materials with strong adhesion ability, better healing promotion, and better spatiotemporal controllability.

Photo-crosslinking reaction using light irradiation is a promising method for the preparation of hydrogels [37]. Photo-crosslinking reaction under light irradiation is a promising method for preparing hydrogels [38]. After the hydrogels are applied to the wound, light is generated under the irradiation of specific visible or ultraviolet light to trigger polymerization, and the hydrogels solidify in situ and change from liquid to solid. Such chemical crosslinking through photoinitiated unsaturated double-bond polymerization can obtain hydrogels with a stable 3D structure and high stiffness and strength [39]. This kind of photo-crosslinking hydrogels has the typical characteristics of “light control”: space–time controllability and nonphysical contact; they can be formed in situ at the time and space we want, and more closely fit the wound, while avoiding the influence of physical factors, such as temperature and PH [40,41]. In addition, the formation of liquid into solid can better fit wet wounds. The photo-crosslinking strategy has been applied in the fields of medicine and engineering, such as the preparation of biomaterials for drug delivery and tissue engineering [34,42,43,44]. In these applications that have been reported, many photo-crosslinking hydrogels have shown good cell compatibility and good cell load, and have higher stiffness and strength; these characteristics determine that the application foreground of photo-crosslinking hydrogels in wound healing is very wide. However, there is no summary report on the application of photo-crosslinking hydrogels in wound healing. Photo-crosslinking hydrogels have the characteristics of in situ retention, spatial and temporal controllability, nonphysical contact, good cell compatibility, and cell loading. Therefore, photo-crosslinking hydrogels have a broader application prospect for clinically more complex irregular wounds and some refractory wounds, such as sinus tract and fistula.

**Figure 1:**
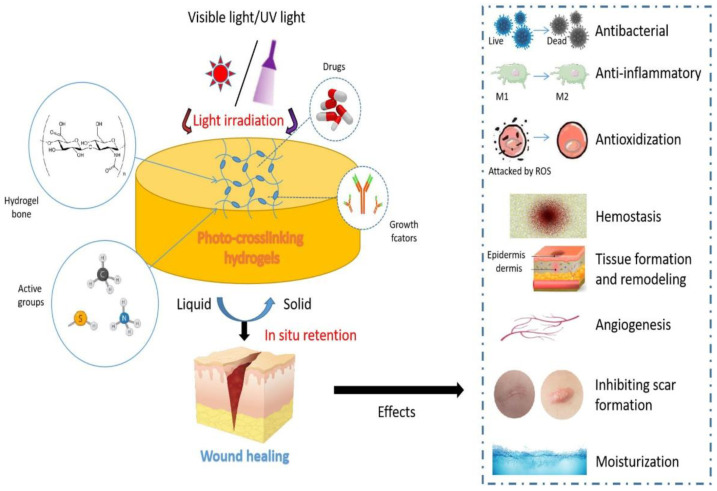
Schematic showing the design and effects of photo-crosslinking hydrogels for wound healing.

In this review, we aim to summarize the current research progress on the application of photo-crosslinking hydrogels in wound healing (Table 1) and mainly introduce the material selection and formation mode of photo-crosslinking hydrogels. The effects of photo-crosslinking hydrogels in promoting wound healing and the animal model used for verification are also discussed (Figure 1). Finally, we analyze the future application prospects of photo-crosslinking hydrogels in the field of wound healing.

## 2. The Design of Photo-Crosslinking Hydrogels for Wound Healing

In general, the formation of photo-crosslinking hydrogels consists of two main methods: photo-initiated polymerization caused by free radicals and photoclick chemistry [68]. Currently photo-crosslinking materials used in wound healing focus on using free radical photopolymerization method preparation. In free radical photopolymerization, the functionalization of the hydrogel bone/hydrogel prepolymer with active groups (such as methacrylate, acrylate) is an essential step: Under light irradiation, the absorbed photons of photoinitiator promote its cleavage, thereby promoting the production of active radical molecules, which will then react with the vinyl bond, leading to the formation of chemical crosslinks between the prepolymers. In addition to this traditional photo-crosslinking preparation method, there are some new photo-crosslinking mechanisms. For example, Zhang et al. used “photo-coupling reaction” to construct hydrogels in situ and used photocage molecules (such as coumarin and o-nitrobenzyl) to release reactive active groups (such as sulfhydryl and aldehyde groups) by photoshear. Further biological coupling reactions (such as Michael addition and Schiff base condensation) are triggered to complete the photo-crosslinking process [45]. The preparation of photo-crosslinking hydrogels has explored several feasible hydrogel materials [69], including polysaccharide substance (hyaluronic acid, alginate, etc.), gelatin, natural fibrin (sericin, silk fibroin), polyethylene glycol (PEG), and natural chitosan polymer. These materials are combined with various active groups (such as methacrylate and acrylate) for photo-crosslinking, and the integrity of the final photo-crosslinking material also depends on the strength of the polymer chain and the density of the crosslinking network in the hydrogel matrix [70].

The toxicity of photo-crosslinking hydrogels remains controversial. The biotoxicity of photo-crosslinking hydrogels is mainly derived from three components: hydrogel prepolymer, light source, and photoinitiator. Most of the hydrogel prepolymers of photo-crosslinking hydrogels are natural macromolecules, and the light source has been evolved from ultraviolet light to visible light (390 and 405 nm) to avoid cytotoxicity [37]. The generation of free radicals through photopolymerization reaction induced by the photoinitiator remains the main concern and bottleneck of the biocompatibility of photo-crosslinking hydrogels. The traditional widely used photoinitiators include Irgacure 2959 (I2959), riboflavin, and so on [71]. Although reactive oxygen species may be produced during the cleavage of these photoinitiators, thus resulting in damage to cells, most of them can be quickly repaired with little impact on cell homeostasis [72]. Moreover, modulations have been carried out to minimize photoinitiator concentration and UV light intensity [73]. Besides, some innovative reaction methods, such as photoshear reaction and photoinitiating thiol-ene reaction, can reduce the cytotoxicity to a greater extent by generating no free radicals or shortening the generation time of free radicals [45].

The following sections systematically introduce the design of photo-crosslinking materials with different active groups.

### 2.1. Hyaluronic Acid

Hyaluronic acid (HA) is a naturally occurring high-molecular-weight glycosaminoglycan [74], which was first isolated from the vitreous of bovine eyes by Karl Meyer and John Palmer in 1934 [75]. There are multiple active groups on the hyaluronic acid backbone [76]. By introducing the active group methacrylate (MA) to chemically modify the hydroxyl group and acid group, the photo-crosslinking hydrogel material methacrylated hyaluronic acid (MAHA) can be obtained [48,51,67]. In addition, the modification of hyaluronic acid with carbohydrazide (CDH) or o-nitrobenzene (NB) produces an imine-crosslinking reaction of the aldehyde group with the hydrazine group under photo-induced conditions, resulting in HA-NB and HA-CDH hydrogels with high mechanical strength and photo-crosslinking properties [45,51]. Fu-jian Xu and his group prepared hydrogels by mixing hyaluronic acid containing azobenzene (Azo) and β-cyclodextrin (CD) to produce photo-crosslinking supramolecular polysaccharide hydrogels with dynamic crosslinking densities based on the interaction between azo groups and β-cyclodextrin groups [46].

### 2.2. Gelatin

Gelatin as a natural hydrogel is a hydrolysate of collagen [77], which is thermally responsive and water soluble [78]. Gelatin methacryloyl (GelMA) can be obtained by the photo-crosslinking reaction of the active group methacrylate and gelatin under a photoinitiator. Compared with other available hydrogel materials, the GelMA hydrogel can meet the requirements of biological function and mechanical strength by various functionalization methods or adding different kinds of biological materials. It combines the advantages of natural and synthetic hydrogels and has been widely used in the biomedical field. There have also been many reports on wound healing [37,47,50,55,57,60,64,65].

### 2.3. Natural Fibrin

Proteins are components of natural tissues and have been widely used in biomedical fields [79]. Silk fibroin (SF), as a common fiber protein, is similar in composition and structure to the natural extracellular matrix (ECM), and has excellent biocompatibility, biodegradability, bioresorption, low immunogenicity, and adjustable mechanical properties [80]. Current studies have shown that silk fibroin can be combined with other polymers to form composite scaffolds, which can further promote cell differentiation, proliferation, and other behaviors, and have better prospects in tissue regeneration [81]. Recent studies have applied photo-crosslinked silk fibroin hydrogels that contain silk fibroin modified with methacrylate and other therapeutic materials to wound healing. When used, methacrylate silk fibroin (MA-SF) endows the mixed hydrogel system with the ability of rapid in situ photo-crosslinking. It also controls the initial release of therapeutic materials to promote wound regeneration, and firmly adheres to the lifting point of the wound, which can also inhibit bacterial aggregation and create a stable and sterile microenvironment [49,58,62]. Sericin (Ser) as another natural fiber protein has also been reported to combine with methacrylate to prepare methacrylated sericin hydrogels (SMH) for wound healing [52].

### 2.4. Natural Chitosan Polymer

Chitosan (CS) is the second most common natural polysaccharide after cellulose, which has excellent biocompatibility, biodegradation, antibacterial ability, and mucosal adhesion [82]. The active amino and hydroxyl groups in chitosan are easy to combine with photoactive groups, so chemical substances such as methylacrylyl and methacrylate are introduced into chitosan to obtain photo-crosslinking hydrogels [83]. At present, the most common method for preparing photo-crosslinking chitosan hydrogels is still grafting methacrylate groups, which have good mechanical properties and biocompatibility [39,41,54,56,63].

### 2.5. Alginate

Alginate is also a kind of polysaccharide substance that can absorb exudation, maintain a moist environment, and reduce bacterial contamination in the treatment of chronic wounds [84]. It has been recently reported that it can also be photo-crosslinked by combining acrylate groups, and the photo-crosslinking hydrogel prepared has high swelling ability, good elasticity, and strength [59].

### 2.6. Polyethylene Glycol (PEG)

Polyethylene glycol (PEG) is a synthetic material consisting of repeated ethylene glycol units [85]. With high hydrophilicity, abundant chemically modified functional groups, and adjustable properties, it is widely used in various biomedical applications [86]. Wei et al. developed a photo-crosslinking adhesive hydrogel based on carboxymethyl chitosan (CMCS) and a photoresponsive polyethylene glycol (PEG) crosslinker by continuous modification of polyethylene glycol with photoresponsive o-nitrobenzyl (NB) and maleimide (Mal) groups, which has good adhesion, antibacterial effect, and antioxidant properties [51].

### 2.7. Decellularized Extracellular Matrix (dECM)

The decellularized extracellular matrixes derived from dermal, cartilage, and adipose tissue may be prepared as decellularized-extracellular-matrix-based photo-crosslinking hydrogels. There have been reports of biologic scaffold materials composed of dECM that can be partially digested with pepsin solubilized and polymerized in situ to form a hydrogel [87]. Due to its inherent ultrastructure and bioactive molecular and chemical composition, dECM can impact cell proliferation, migration, and differentiation [88,89]. In recent years, dECM-based photo-crosslinking hydrogels have been mixed with other materials into multifunctional hydrogel in terms of antibacterial and antioxidant activities, with their limitations of relative weaker mechanical strength [90,91,92].

## 3. Effects of Photo-Crosslinking Hydrogels in Wound Healing

Taking into account the promotion in wound healing possessed by hydrogel itself, photo-crosslinking may provide hydrogel a stable 3D structure with preferable mechanical strength and wet-tissue adhesive capacity without sutures. Moreover, the photoinitiated triggering modulation is spatiotemporally controllable in a simple, precise, and noncontact manner. The specific two-step procedure could effectively avoid the formation of dead space in different kinds of complex nonhealing wounds, followed by the formation of stable solid hydrogel to isolate the defect from the external environment. All the aforementioned factors contribute to the protection of the macromolecular hydrogel bone materials along with the loaded drugs and bioactive substances, thus avoiding any secondary damage to wound tissue while facilitating patients’ daily activities and improving their quality of life (QOF). Here is the specific effect of photo-crosslinking hydrogels to promote wound healing.

### 3.1. Antibacteria

When wounds are generated, microorganisms are easy to invade and cause infection, which is a prominent factor affecting wound healing [10]. Therefore, antibacterial ability is very important for wound healing materials. The photo-crosslinking hydrogel has good in situ curing characteristics and excellent cell loading. The antibacterial effect is achieved through the inherent antibacterial properties of the photo-crosslinking scaffolds and through the loading of therapeutic ions or metal nanomaterials. Chitosan and alginate are the main photo-crosslinking materials with antimicrobial properties. Previous studies have reported that photo-crosslinking hydrogels containing quaternary ammonium chitosan exhibit good antimicrobial properties [52,56]. However, the antibacterial properties of photo-crosslinking materials alone are not excellent enough, so the addition of antimicrobial active ingredients is the most common solution. Pang et al. reported a composite hydrogel prepared by mixing the therapeutic ion Cu^2+^ with borosilicate (BS) and photo-crosslinking methacrylate silk fibroin (MA-SF). When used, the composite system could thoroughly spread to the whole wound through in situ photo-crosslinking, firmly fit the wound, and protect the wound from external pollution. It can also further promote wound regeneration by releasing therapeutic ions [49]. In addition, Tang et al. encapsulated silver nanoparticles (AgNPs) in hydrogels, which endow the material with long-term broad-spectrum antibacterial activity by continuously transferring silver ions [48]. Yao et al. produced a ZN-MOF encapsulated methacrylate hyaluronic acid hydrogel, and zinc ions also have the ability to destroy the bacterial envelope [50]. In addition, the hydrogel combining gallium ion (Ga^3+^) with alginate has the effect of destroying bacterial iron metabolism and exhibits good antibacterial activity [61].

### 3.2. Anti-Inflammatory

The first stage of wound healing is the inflammatory phase. After injury, inflammatory pathways and components of the immune system are recruited to remove necrotic tissue [4]. Excessive and prolonged inflammation can lead to poor wound healing, such as scarring. Limited inflammatory response is conducive to wound healing, so the idea of preparing anti-inflammatory hydrogels is to give them the ability to reduce the number of macrophages and neutrophils, reduce proinflammatory factors, improve inflammatory inhibitory factors, and promote macrophage polarization [27].

Qi et al. designed a photo-crosslinking sericin hydrogel (SMH), and evaluated inflammatory cells (CD68-stained macrophages and myeloperoxidase (MPO)-stained neutrophils) were approximately twofold lower than conventional treatment. These results suggest that sericin hydrogels can inhibit inflammatory responses during healing, but the specific mechanism has not been clarified [53].

Reducing proinflammatory chemokine production is a common strategy to reduce inflammation. In one study, a photo-crosslinking hydrogel mixed with thiol–acrylate and alginate/poloxamer activated human keratinocyte proliferation and produced anti-inflammatory effects by inhibiting the extracellular ERK-NF-KB-TNF-α signaling pathway. Its anti-inflammatory test results showed a substantial decrease in the proinflammatory chemokine TNF-α, comparable to the results obtained with the anti-inflammatory drug dexamethasone treatment [59].

M1 macrophages promote the development of inflammation, and M2 macrophages inhibit M1 macrophages by secreting anti-inflammatory cytokines, such as IL10. Therefore, regulating the polarization of macrophages and realizing the effective transformation from M1 to M2 is also a feasible strategy to reduce the inflammatory response and promote wound healing [49,50,62,67]. Metformin (Met) is a classic oral hypoglycemic drug. A large number of previous studies have shown that metformin has good immunomodulatory properties in vivo and in vitro, and exogenous metformin can induce macrophage polarization to the anti-inflammatory phenotype M2 [93,94]. An immunomodulatory drug (M@M-Ag-Sil-MA) has been designed in recent studies, which was further loaded with antibacterial silver nanoparticles (AgNPs), and metformin loaded with mesoporous silicon nanoparticles (MET@MSNs) using photo-crosslinking methacrylate silk fibroin hydrogel (Sil-MA) as a scaffold. Both in vitro and in vivo tests confirmed that this material can induce the effective transformation of macrophages M1 to M2, thereby reducing the inflammatory response for wound healing [58].

### 3.3. Anti-Oxidization

Many studies have demonstrated that the presence of excessive reactive oxygen species (ROS) can hinder the process of wound healing [95,96,97]. The sustained inflammatory response during wound healing leads to the accumulation of a large number of oxygen free radicals, which exceeds the antioxidant capacity of cells, thus preventing the transition of wounds from the inflammatory phase to the proliferative phase [98]. Therefore, the use of antioxidant materials in wound healing can effectively promote cells to maintain REDOX balance, thus accelerating wound healing. Huang et al. introduced tofu into GelMA hydrogel by the photo-crosslinking method for the first time, and found that the antioxidant activity of the mixed hydrogel strengthened with the increase in tofu [50]. In another study, a photo-crosslinking hydrogel mixed with the natural antioxidant glycyrrhizin (GA) showed significant antioxidant activity, with a free radical scavenging capacity of over 90% [62]. In addition, some stable free radicals can be introduced into photo-crosslinking hydrogels as antioxidants to remove excess oxygen free radicals. For example, grafting phenol groups with good antioxidant properties onto photo-crosslinking carboxymethyl chitosan (CMCS) bones can give hydrogels effective and long-term antioxidant properties [52].

### 3.4. Hemostasis

The inflammatory phase of wound healing requires the coagulation cascade to stop bleeding, which includes vasoconstriction, platelet thrombosis, and blood coagulation [4,5]. Photo-crosslinking hydrogels can be used as tissue adhesives to control wound bleeding due to their in situ curing and sealing properties [99]. For example, the tissue adhesive methacrylate hyaluronic acid–polyacrylamide (MHA–PAAm) hydrogel was verified to have good hemostatic ability in the model of chronic wound bleeding and acute massive bleeding simulating clinical scenes. SEM electron microscope observation showed that many red blood cells appeared on the surface of the hydrogel. This suggests that the excellent hemostatic performance of MHA–PAAm hydrogel is due to the electrostatic attraction between its positive charge and the negative charge of red blood cells, which promotes the formation of platelet thrombosis [48]. In addition, because of the hemostatic effect of hydrogel skeleton chitosan and good tissue adhesion ability, CSG-PEG/DMA6/Zn photo-crosslinking hydrogel can be photo-crosslinked in situ and closely fit the wound site, providing a stable gel network as a physical barrier to accelerate blood coagulation. Its hemostatic function was proved to be superior to the traditional chitosan wound dressing previously reported in the mouse liver hemorrhage model and the mouse tail amputation model [41].

### 3.5. Tissue Formation and Remodeling

The proliferative phase is particularly important in wound healing, and granulation tissue composed of macrophages, fibroblasts, and endothelial cells is a marker of the proliferative phase. In addition, re-epithelialization is essential for rebuilding tissue integrity [5]. Loading various growth factors into photo-crosslinking hydrogels is an effective way to improve the efficiency of wound healing [68], which can improve granulation tissue formation, promote collagen deposition, and accelerate the process of re-epithelialization. For example, Li et al. added endogenous vasoconstrictor peptide endothelin-1 (ET-1) into photo-crosslinking gelatin methylacrylyl (GelMA) hydrogel. ET-1 was encapsulated in the crosslinked hydrogel network through intermolecular hydrogen bond interaction, which improved stability and effectively avoided oxidative degradation. Further in vivo experiments showed that GELMA-ET-1 hydrogel significantly accelerated the formation of new blood vessels, collagen deposition, and epithelial regeneration [47]. In addition, basic fibroblast growth factor (B-FGF), which promotes tissue regeneration, has also been reported to be combined with photo-crosslinking materials to form gelatin photo-crosslinking hydrogels with rapid wound adhesion, wet tissue surface adhesion, and long-term bFGF release. This material can promote cell migration and improve wound healing and flap formation [37].

### 3.6. Promoting Angiogenesis

Angiogenesis plays an important role in wound healing by facilitating the transport of nutrients and oxygen to the lesion site [100,101]. Direct loading of angiogenic substances into photo-crosslinking hydrogels is the most common method. Differin (DFO) is an FDA-approved iron chelator for clinical use. Previous studies have shown that DFO can accelerate the formation of new blood vessels under normal and pathological conditions by upregulating the expression of HIF-1α and its downstream gene VEGF [102,103,104]. Chen et al. prepared gelatin hydrogels loaded with DFO using physical mixed photo-crosslinking technology. At the early stage of injury, the photo-crosslinking hydrogels released wrapped DFO to promote the formation of a vascular network, deliver a sufficient amount of oxygen and nutrients to the wound area, and promote the proliferation of granulation and epithelial tissues [66]. In addition, loading the photo-crosslinking hydrogel network with exosomes or extracellular vesicles (EVs) that secrete growth factors has proved to be a feasible strategy. As natural carriers, EVs can directly transfer endogenous bioactive molecules to recipient cells to play therapeutic functions [105,106]. In a study by Wang et al., extracellular vesicles derived from epidermal stem cells (ESCs) were loaded with the HIF-1α stabilizer VH298 and encapsulated in photo-crosslinking GelMA hydrogel. This material effectively promotes wound healing by activating the HIF-1α signaling pathway to locally enhance blood supply and angiogenesis [64].

### 3.7. Inhibiting Scar Formation

The final remodeling stage of wound healing can last for several years. At this stage, extracellular matrix components change to close to normal tissue architecture, the wound margin physically contracts, and granulation tissue stops growing due to apoptosis [6]. Abnormalities in either process may lead to the formation of keloids and hypertrophic scars. Transforming growth factor-β (TGF-β) plays a pleiotropic role in wound healing by regulating cell proliferation and migration, extracellular matrix production, and immune response [107,108]. Therefore, focal blocking of TGF-β pathway expression has been considered an effective therapeutic target for the treatment of abnormal wound formation and scar hyperplasia. In a study by Zhang et al., pulse-released PLGA (polylactic acid glycolic acid)-NB capsules containing a TGF-β inhibitor (SB-431542) were loaded onto photo-crosslinking HA-NB and HA-CDH hydrogels. They have been proved to inhibit scar formation in a mouse full-thickness skin defect model, rabbit ear hypertrophic scar model, and pig skin defect model [45]. In addition, Chao et al. developed a photo-crosslinking sericin hydrogel (SMH), which can prevent scar tissue formation by regulating the expression of TGF-β1 and TGF-β3 and recruit mesenchymal stem cells to the injury site to regenerate skin appendages [53]. In conclusion, these photo-crosslinking hydrogel designs utilizing TGF-β pathway blockade show a potential for scarless wound healing.

### 3.8. Water Retaining Capability

Long-term moisturization is also crucial for promoting wound healing. Compared with treatment in a dry environment, a moist environment can protect the formation of new granulation tissue, promote the formation of wound epithelium, and lead to less scar formation [109]. However, traditional hydrogels tend to evaporate in an open environment, which reduces their tensile properties and water retention [110]. Therefore, the manufacture of hydrogels with long-term mechanical stability is particularly critical for wound moisture. A recent study introduced tea polyphenol (TP)/glycerol (TG) into a covalent crosslinking network consisting of photo-crosslinking N-allylglycine (NAGA), GelMA, and Laponite so that a multifunctional hydrogel with high water retention, long-term mechanical stability, antibacterial and antioxidant properties was synthesized [57]. The glycerol/water solvent delayed the diffusion of tea polyphenols in the hydrogel and formed a uniform network, which was conducive to water retention.

## 4. Application of Animal Models for Wound Healing

**Figure 2:**
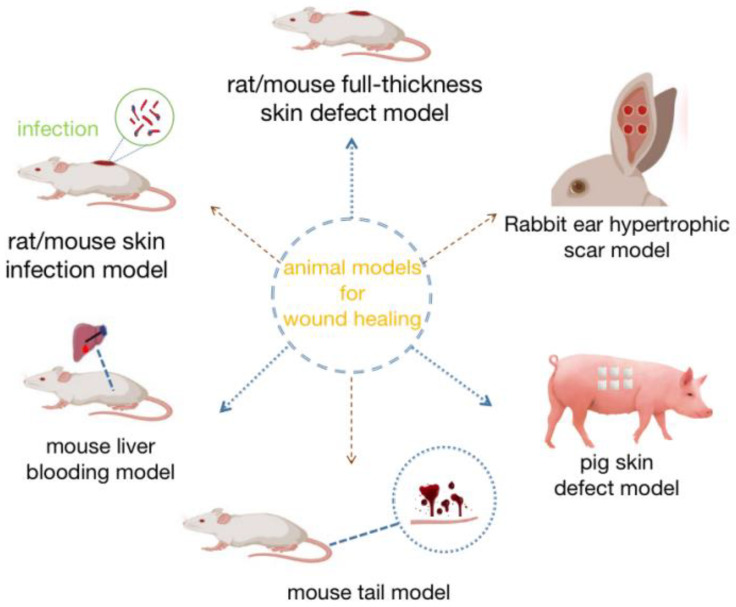
Animal models used in wound healing.

Animal models have long been considered the best nonhuman models for wound healing. In vitro assays cannot fully recapitulate biological conditions, such as immune response, healing, and disease; thus, animal models are necessary for scientific evaluation [111,112]. Due to the anatomical and physiological differences between various animal species including humans, both small and large animal studies have their advantages and limitations [113]. In terms of studying the effect of photo-crosslinking hydrogels on wound healing, most of the studies were conducted on rodents, followed by rabbit and pig models (Figure 2). Zebrafish and drosophila are novel wound healing models [114,115], and there have been no reports on the verification of the use of photo-crosslinking hydrogel, but their genetic traceability has the potential to promote the exploration of more mechanisms of wound healing [116]. Several animal models used to evaluate the effect of photo-crosslinking hydrogels on wound healing are described below.

### 4.1. Rat/Mouse Full-Thickness Skin Defect Model

The rat/mouse full-thickness skin defect model is the most commonly used model to verify the mechanism of wound healing. The rat/mouse model has low application cost, easy operation, many transgenic lineages, and many detection technologies [117,118]. We can evaluate the effect of photo-crosslinking hydrogel on wound healing by various means, such as histology, immunohistochemistry, fluorescence staining, and ELISA detection of rat/mouse samples. For example, Zhao et al. constructed a full-thickness skin defect model in rats. By observing the wound area, the collagen deposition in skin tissue samples, and the expression of TGF-β detected by ELISA, it was finally verified that the photo-crosslinking hydrogel had good healing efficiency in vivo [46]. In addition, the level of inflammatory reaction can be detected in the rat/mouse full-thickness skin defect model [45,50,53], and the detection results also show that anti-inflammatory is an important role of photo-crosslinking hydrogel in promoting wound healing.

### 4.2. Bleeding Model (Mouse Tail Model and Mouse Liver Bleeding Model)

In vitro tests, the photo-crosslinking hydrogels have been proved to have good tissue tightness. Therefore, it is necessary to construct bleeding models simulating clinical scenarios to test the hemostatic effect. Tang et al. and Yang et al. both used the mouse tail model and the mouse liver hemorrhage model to simulate chronic bleeding and acute massive bleeding in wounds, respectively [41,48]. In addition, the rabbit liver injury model has also been applied to simulate the visceral bleeding that often occurs during surgery [39]. The final test results showed that the photo-crosslinking hydrogels prepared by the above three studies had good hemostatic performance. This suggests that the photo-crosslinking hydrogel has good tissue adhesion ability, which can tightly adhere to the wound and provide a stable hydrogel network as a physical barrier to accelerate coagulation, which also shows the potential of the photo-crosslinking hydrogel as a tissue sealant.

### 4.3. Rabbit Ear Hypertrophic Scar Model

Although rodent provides a powerful and rapid model for wound healing, the dorsal skin of rats/mice is loose and lacks tension [119]. Additionally, only completing the healing speed through contraction is faster, but it not easy to form a scar [120,121]. In contrast, the skin of rabbit ears is tight, and the wound does not heal quickly due to contraction, and delayed re-epithelialization leads to abnormal scar growth similar to hypertrophic scar formation in humans [122,123]. Zhang et al. used the reproducible and quantifiable rabbit ear hypertrophic scar model established by Mustoe et al. [122], and applied its photo-crosslinking hydrogel loaded with PLGA capsule to cover the wound until the wound was fully epithelialized. The results of wound section staining and the scar bulge index (SEI) all suggest that the photo-crosslinking hydrogel prepared in this study has a significant effect on inhibiting scar growth [45].

### 4.4. Pig Skin Defect Model

The skin structure and hair density of pigs are most similar to those of humans. As an animal model, pigs can reproduce the unique pathological conditions of humans, such as hypertrophic scars [124]. However, in terms of disadvantages, pigs are expensive and genetically heterogeneous and require a high level of operation [125]. More importantly, pigs are not well characterized at the cellular and physiological levels compared with rodents, and specific porcine reagents, such as antibodies and growth factors, remain difficult to apply. Therefore, although the pig skin defect model is very close to that of human, only some ordinary histological observations can be carried out, and it is difficult to explore the deeper mechanism.

In addition to the several commonly used animal models listed above, rat/mouse skin infection models are widely used to detect in vivo antimicrobial levels in photo-crosslinking hydrogel [48,49,51,61]. In addition, there are some special wound model constructions, such as the chronic wound healing model constructed by the diabetic skin defect model [37,49,58,62,64,65], the rat/pig mucosal defect model for detecting the wound healing of oral ulcer [50], and the rabbit focal corneal defect model for detecting the corneal healing [60].

## 5. Conclusions and Future Perspectives

Photo-crosslinking hydrogels are prepared by visible light/UV light irradiation reaction, which have the advantages of controllable time and space, high polymerization efficiency, not being affected by temperature conditions, curing in situ, and easiness of control and use. Current studies have shown that photo-crosslinking hydrogels exhibit excellent cell compatibility, good cell loading, and higher stiffness and strength, which determine the broad application prospect of photo-crosslinking hydrogels in wound healing. In this review, we summarize the effects of photo-crosslinking hydrogels in promoting wound healing. Due to the highly alike biomechanical characteristics to the ECM of photo-crosslinking hydrogel, macromolecular hydrogel bone materials along with the loaded drugs and bioactive substances can maximize the role of promoting wound healing, such as antibacterial, anti-inflammatory, antioxidation, hemostasis, tissue remodeling, angiogenesis, inhibition of scar hyperplasia, and moisturizing. It has the potential to become a variety of clinical products, such as wound dressings (especially for irregular wounds), antibacterial dressings, tissue adhesives, tissue sealants, rapid hemostatic agents, scar inhibitors, and corneal substitutes. However, there have not been a lot of reports on human experiments at present. As a potential excellent medical material, the photo-crosslinking hydrogel needs to achieve clinical transformation as soon as possible. The toxicological, safety, and efficacy effects of the hydrogel should be determined in preclinical tests, and a well-controlled randomized clinical trial should be conducted to demonstrate the true application potential of the hydrogel. We anticipate that in the near future, clinically available and commercially accessible photo-crosslinking hydrogels will be widely used in the fields of general surgery, oral and maxillofacial surgery, orthopedics and plastic surgery, and so on, particularly in clinical applications when surgeons demand temporary coverage for complex, irregular-shaped, and nonhealing wounds. To the best of our knowledge, all the aforementioned advantages may provide great pliability and flexibility during patients’ daily activities, thus resulting in a better QOF for such patients suffering from oral mucosa ulcer, pressure ulcers, sinus, or full-thickness defect. Moreover, the combination of endoscope, arthroscopy, and so on with photo-crosslinking hydrogels also bring modifications and evolutions to traditional surgical procedures.

## Figures and Tables

**Table 1:** Photo-crosslinking hydrogels for wound healing.

Hydrogels	Design		Effects	Animal Models	Application Prospect	Ref.
	Hydrogel Backbones	Chemical Modification				
Poly-lactic-co-glycolic acid (PLGA) imine-crosslinking hydrogels	Hyaluronic acid (HA)	o-Nitrobenzene (NB), carbohydrazide (CDH)	TGF-β inhibitor	Murine skin wound repair model Rabbit ear and porcine skin wounding model	Tissue adhesive, scar inhibitor	[45]
Photoresponse supramolecular hyaluronic acid hydrogels	HA	Azobenzenes (Azo), β-cyclodextrin (CD)	EGF delivery (improving granulation tissue formation, high growth factor levels, and angiogenesis)	Rat full-thickness skin defect model	New smart dressings	[46]
Photo-crosslinking GelMA-ET-1 hydrogels	Gelatin (Gel)	methacryloyl (MA)	ET-1 delivery (accelerating new blood vessel formation, collagen deposition, and re-epithelialization)	Rat full-thickness skin defect model	Wound dressings	[47]
Methacrylated hyaluronan–polyacrylamide (MHA–PAAm) hydrogels	HA	MA	Antibacterial and hemostatic activities Tissue formation (enhancing wound granulation tissue formation, vascular tissue formation, and collagen formation, as well as alleviating inflammation)	Rat tail amputation and liver injury model Rat wound infection models	Skin adhesive	[48]
SF-MA-BS hydrogels	Silk fibroin (SF)	MA	Antibacterial Angiogenesis by restoring the HIF-1α pathway Transformation of macrophages to M2 type	Rat streptozotocin-induced diabetic wound repair model	Diabetic wound dressings	[49]
Tofu-based hybrid GelMA hydrogels	Gel	MA	Antioxidant Transformation of macrophages to M2 type	Rat full-thickness skin defect model	Antioxidant	[50]
Cyclic o-nitrobenzyl-modified hyaluronic acid (HA-CNB) hydrogels	HA	Cyclic o-nitrobenzyl (CNB)	Transformation of macrophages to M2 type	Rat and pig mucosa defect model	Oral mucosal adhesive	[51]
Zn-MOF encapsulated methacrylated hyaluronic acid (MeHA) hydrogels	HA	MA	Antibacterial tissue formation (promoting angiogenesis, deposition of collagen, and reduced inflammation)	Rat full-thickness skin defect model	Microneedles (MNs)	[51]
HB-QCMCS/PEG hydrogels	Polyethylene glycol (PEG)	NB	Antibacterial Antioxidant	Rat full-thickness skin defect model	Tissue adhesive	[52]
Photo-crosslinked gelatin hydrogels	Gel	MA	b-FGF delivery (improving granulation tissue formation)	Rat diabetic wound repair model	Irregular wound healing	[37]
Methacrylated sericin (SerMA) hydrogels	Sericin (Ser)	MA	Antibacterial Inhibiting inflammation Promoting angiogenesis TGF-β inhibitor	Mouse full-thickness skin injury model	Skin wound dressing	[53]
H (Non-P), H (P), and H (P + T) multifunctional bioadhesive hydrogels	Carboxymethyl chitosan (CMCS)	MA	Antibacterial Antioxidant Promoting angiogenesis	Rat infected full-thickness skin defect model, Rabbit liver injury model	First-aid hemostasis, wound dressing	[39]
Triple crosslinked poly (vinyl alcohol)/methacrylate kappa-carrageenan/chitooligosaccharide hydrogels	Kappa-carrageenan (κ-Ca)	MA	Antibacterial Antioxidant	Mouse full-thickness skin injury model	Wound dressing	[54]
GelMA-dHAMMA composite hydrogels	Gelatin	MA	Promoting angiogenesis Tissue formation (deposition of collagen)	Rabbit skin defect model	Skin substitute	[55]
PQB2 hydrogels (a double-crosslinked self-healing antibacterial hydrogel)	Quaternized methacryloyl chitosan (QMC)	Methacrylic anhydride	Antibacterial inhibiting inflammation	Mouse infected full-thickness skin defect model	Dressing for promoting infectious wound healing	[56]
CSG-PEG/DMA/Zn hydrogels	Chitosan (CS)	Methacrylate anhydride	Antibacteria Antioxidant Hemostasis inhibiting inflammation	Rat tail amputation and liver injury model Mouse infected full-thickness skin defect model	Drug-resistant bacteria infected skin wound dressing	[41]
NGLG20/TG hydrogels	Gel	MA	Water-retaining capability Antibacteria Antioxidant Tissue formation (deposition of collagen)	Rat full-thickness skin defect model	Wound dressing	[57]
M@M–Ag–Sil-MA hydrogels	Silk fibroin	MA	Antibacterial Transformation of macrophages to M2 type Promoting angiogenesis	Rat diabetic wound repair model	Engineered nanodressing	[58]
Degradable alginate/poloxamer hydrogels	Alginate	Acrylate	Inhibiting inflammation	——	Wound dressing	[59]
Thiol–acrylate hydrogels	Gel	Acrylate anhydride, cysteamine	Tissue formation (exhibiting epithelial wound coverage)	Focal corneal injury rabbit model	Corneal substitutes	[60]
Gallium (III)-mediated dual-crosslinked alginate hydrogels	Alginate	Acrylate	Antibacterial TGF-β inhibitor Tissue formation (promoting angiogenesis, deposition of collagen, and reduced inflammation) Water retaining capability	Mouse bacteria-infected wound repair model	Wound dressing	[61]
SF/GA/Zn hybrid hydrogels	Silk fibroin	MA	Inhibiting inflammation transformation of macrophages to M2 type	Rat diabetic wound repair model	Diabetic wound dressing	[62]
MCS/PEGDF/PEGDA/DMA hydrogels (CF gel for short)	Maleic anhydride modified chitosan (MCS)	MA	——	Rat full-thickness skin defect model	Tissue sealant	[63]
GelMA/Gel-VH-EVs hydrogels	Gel	MA	Promoting angiogenesis tissue formation (deposition of collagen)	Mouse diabetic wound repair model	Diabetic wound dressing	[64]
DFO–Gelma hydrogels	Gel	MA	Promoting angiogenesis Tissue formation (granulation tissue remodeling and epithelial crawling)	Rat diabetic wound repair model	Diabetic wound dressing	[65]
Curcumin complexed β-CD loaded glycol chitosan hydrogels	Glycol chitosan (GC)	Glycidyl methacrylate (GM)	Promoting angiogenesis Inhibiting inflammation Tissue formation (granulation tissue remodeling and epithelial crawling)	Mouse full-thickness skin defect model	Wet dressing	[66]
HA-MA-NB (HNM) hydrogels	HA	MA	Transformation of macrophages to M2 type Promoting angiogenesis	Rat diabetic wound repair model	Wound dressing	[67]

## Data Availability

Not applicable.
